# High-altitude-induced alterations in intestinal microbiota

**DOI:** 10.3389/fmicb.2024.1369627

**Published:** 2024-05-09

**Authors:** Dan Liu, Dan Chen, Jian Xiao, Wei Wang, Li-Juan Zhang, Hui Peng, Chuan Han, Hao Yao

**Affiliations:** ^1^Department of Endocrinology, General Hospital of the Chinese People’s Liberation Army Western Theater, Chengdu, Sichuan, China; ^2^Department of Hematology and Hematopoietic Stem Cell Transplantation Center, General Hospital of the Chinese People’s Liberation Army Western Theater, Chengdu, Sichuan, China

**Keywords:** high altitude (low air pressure), acute mountain sickness (AMS), intestinal microbiota, Han adolescent, Tibet (China)

## Abstract

In high-altitude environments characterized by low pressure and oxygen levels, the intestinal microbiota undergoes significant alterations. Whether individuals are subjected to prolonged exposure or acute altitude changes, these conditions lead to shifts in both the diversity and abundance of intestinal microbiota and changes in their composition. While these alterations represent adaptations to high-altitude conditions, they may also pose health risks through certain mechanisms. Changes in the intestinal microbiota induced by high altitudes can compromise the integrity of the intestinal mucosal barrier, resulting in gastrointestinal dysfunction and an increased susceptibility to acute mountain sickness (AMS). Moreover, alterations in the intestinal microbiota have been implicated in the induction or exacerbation of chronic heart failure. Targeted modulation of the intestinal microbiota holds promise in mitigating high-altitude-related cardiac damage. Dietary interventions, such as adopting a high-carbohydrate, high-fiber, low-protein, and low-fat diet, can help regulate the effects of intestinal microbiota and their metabolic byproducts on intestinal health. Additionally, supplementation with probiotics, either through dietary sources or medications, offers a means of modulating the composition of the intestinal microbiota. These interventions may offer beneficial effects in preventing and alleviating AMS following acute exposure to high altitudes.

## 1 Introduction

The high-altitude environment (defined medically as regions above 3,000 m altitude) is characterized by low oxygen pressure, cold climate, high wind speeds, high evaporation rates, high radiation, and variable weather conditions (Robles and Guarner, 2013). These factors can have various adverse effects on individuals, leading to physiological discomforts and health challenges upon initial exposure (Robles and Guarner, 2013). Upon rapid ascent to high altitudes, the environmental stressors, particularly low oxygen levels, can compromise the integrity of the gastric mucosal barrier function. Consequently, some individuals may experience increased gastric acid secretion, resulting in acute gastrointestinal injury (Robles and Guarner, 2013; Yadav et al., 2016). The intestine serves as the largest reservoir of symbiotic bacteria in the human body (Clyde, 2019). The intestinal microbiota, a critical component of the intestinal microenvironment, plays a pivotal role in establishing and maintaining a stable nutritional balance microenvironment (Clyde, 2019). Moreover, it interacts with the human host, influencing immune function, metabolism, and disease susceptibility (Festi et al., 2011). Mounting evidence underscores the significance of intestinal microbiota in modulating human health and disease outcomes. The diversity of intestinal microbiota reflects the outcome of host selection and coevolution, which is subject to the influence of various factors.

In recent years, there has been increasing recognition of the significant impact of environmental factors on the intestinal microbiota (Ma et al., 2019; Wang et al., 2022). Specifically, research has highlighted the role of the high-altitude hypoxic environment in shaping the composition and structure of the intestinal microbial community (Bai et al., 2022; Wang et al., 2022). However, our understanding of the precise effects of extreme environments, such as high altitudes, on the intestinal microbiota remains incomplete. Emerging evidence suggests that exposure to hypobaric hypoxia at high altitudes induces changes in the intestinal microbiota (Ma et al., 2019). Yet, it remains challenging to discern whether these alterations in microbial composition are primarily reflective of host physiological adaptations or whether they contribute directly to the onset or exacerbation of high-altitude illnesses. The adverse conditions present in high-altitude environments have driven natural selection among populations (Bai et al., 2022). Factors such as hypobaric hypoxia, low temperatures, and high radiation not only impact organismal reproduction and survival but also accelerate population-level evolution and physiological adaptations (Hu et al., 2022). These conditions may also precipitate high-altitude reactions or illnesses in susceptible individuals, potentially leading to dysbiosis in the intestinal microbiota (Lan et al., 2017; Zuo et al., 2022).

Moreover, the intestinal microbiota acts as a critical regulatory factor in response to high-altitude environments, influencing the adaptive processes of the body (Zhao et al., 2022; Zuo et al., 2022). Various enzymes, metabolic products, and signaling molecules produced by the intestinal microbiota may modulate the response of the body to high-altitude conditions through intricate signaling pathways.

Currently, there is a paucity of research elucidating the specific mechanisms by which high-altitude hypoxia influences the structure of the intestinal microbiota (Li et al., 2022; Zhao et al., 2022). Understanding these mechanisms is essential as it can provide insights into the ways hypobaric hypoxia impacts gastrointestinal microbiota damage. Further exploration into the effects of rapid ascent to high altitudes on the intestinal microbiota and its implications for gastrointestinal health is warranted to enhance the wellbeing of individuals traveling to such environments.

## 2 The changes in intestinal microbiota after exposure to high-altitude environments

### 2.1 Changes in intestinal microbiota following prolonged exposure to high-altitude environments

#### 2.1.1 Changes in animal intestinal microbiota after long-term exposure to high-altitude environments

**Figure 1:**
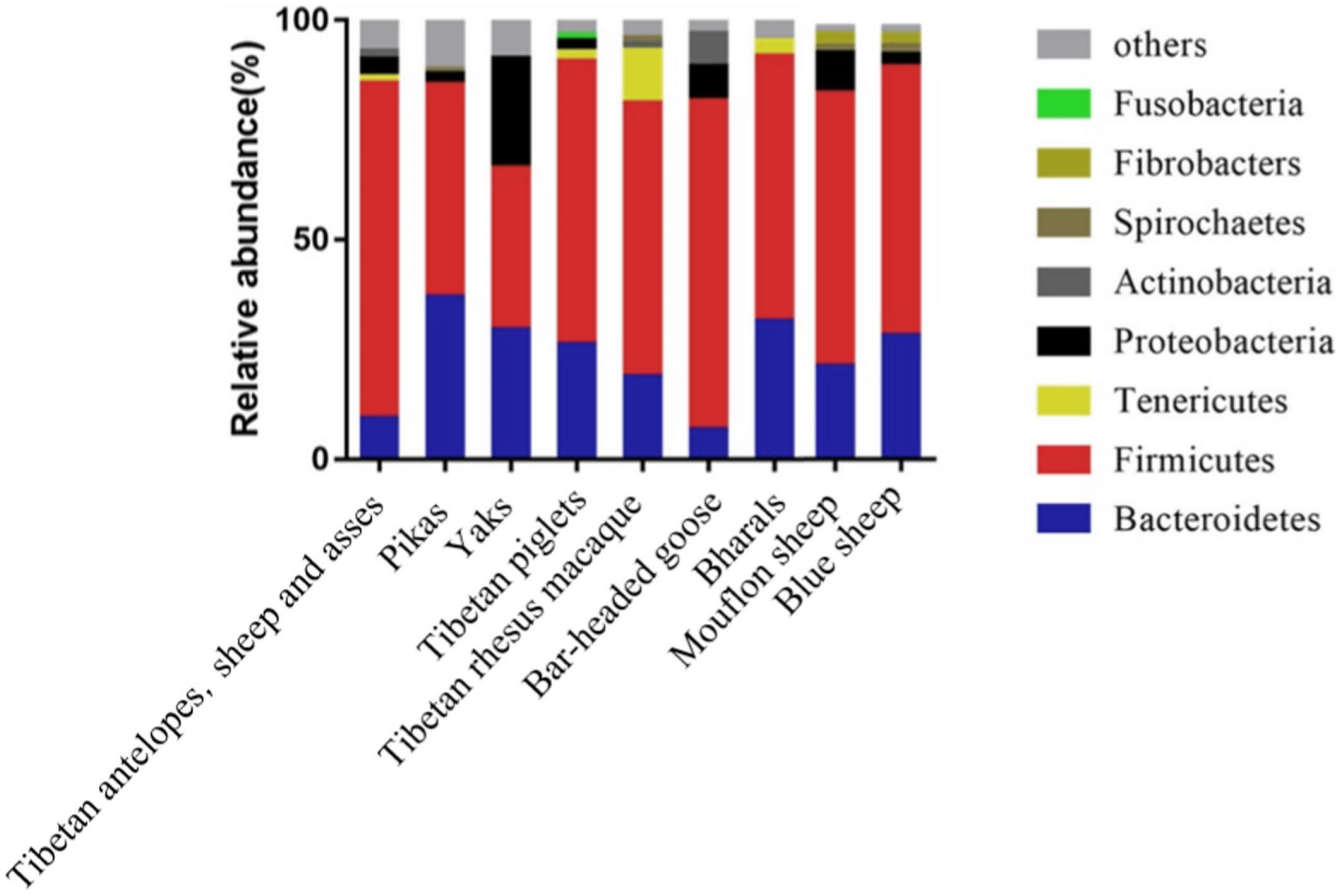
Composition of intestinal microbiota in mammals inhabiting high-altitude regions.

Compared to mammals living in plain areas, those inhabiting high-altitude regions exhibit noticeable alterations in their intestinal microbiota. As depicted in Figure 1, mammals such as Tibetan antelopes, Tibetan wild donkeys, yaks, plateau pikas, Tibetan pigs, Tibetan macaques, yaks, bar-headed geese, bighorn sheep, mouflons, and blue sheep, whether dwelling at high or low altitudes, predominantly harbor the phyla Firmicutes and Bacteroidetes (Li and Zhao, 2015; Zhang et al., 2016; Li et al., 2018; Zhao et al., 2018; Ma et al., 2019; Liu et al., 2021). However, the composition and structure of intestinal microbiota vary across different altitudes. For instance, at an altitude of 4,331 m, the relative abundance of Firmicutes and Bacteroidetes in pikas’ intestines significantly differs from that at 3,694 m and 3,856 m (Obregon-Tito et al., 2015). Studies on wild ungulates at high altitudes (Yaks, Tibetan wild donkeys, Tibetan antelopes, and Bighorn sheep) also reveal that Firmicutes/Bacteroidetes ratios are higher than those in low-altitude ungulates, with Firmicutes and Proteobacteria being the most enriched phyla in the intestinal microbiota of high-altitude ungulates (Table 1; Guo et al., 2014; Du et al., 2022; Wang et al., 2022). In particular, the Firmicutes/Bacteroidetes ratio (F/B ratio) in the intestinal microbiota of high-altitude mammals such as Plateau pikas, Tibetan macaques, Tibetan antelopes, Mouflons, and Blue sheep exceeds that of low-altitude mammals. Given that Firmicutes and Bacteroidetes primarily aid in digesting and absorbing nutrients, breaking down carbohydrates and proteins, respectively, a higher F/B ratio may facilitate efficient energy extraction from the diet, thus maintaining energy homeostasis and core body temperature in harsh environments (Zhao et al., 2018; Zeng et al., 2020).

**Table 1:** Research progress on intestinal microbiota of animals and humans exposed to plateau environment.

	Research subject	Study time	References
Study on indigenous animal gut microbiota adaptation to plateau	Variable temperature snake	2018	Li et al. (2018)
	Chinese rhesus monkey	2018	Hu et al. (2022)
	Yak	2016	Zhang et al. (2016)
		2022	Wang et al. (2022)
	Tibetan antelope	2016	Zhang et al. (2016)
		2022	Wang et al. (2022)
	Equus kiang	2022	Wang et al. (2022)
	Blue sheep	2022	Wang et al. (2022)
	Tibetan chicken	2022	Du et al. (2022)
	Plateau zokor	2023	Hu et al. (2023)
	Tibetan pig	2020	Zeng et al. (2020)
	Plateau pika	2023	Hu et al. (2023)
Study on intestinal microbiota of high-altitude population adaptation to plateau	Tibetan and Han populations in low- and high-altitude areas	2015	Li and Zhao (2015)
	Tibetan population	2016	Greenhill et al. (2015) and Li et al. (2016)
	Tibetan population in low- and high-altitude areas	2015	Li and Zhao (2015)
		2015	Greenhill et al. (2015) and Das et al. (2018)
		2020	Guo et al. (2014) and Zeng et al. (2020)
	Indian population in high-altitude areas	2018	Tandon et al. (2018)
	Indian population in low- and high-altitude areas	2018	Das et al. (2018)
	Papua New Guinean population in low- and high-altitude areas	2015	Greenhill et al. (2015) and Das et al. (2018)
Study on intestinal microbiota adaptation to acute plateau exposure	Mountaineers	2005	Costea et al. (2018)
	Mice	2018	Zhang et al. (2018)
	Rats	2015	Zhang et al. (2015)
		2018	Zhang et al. (2018)
Study on acute plateau sickness and intestinal microbiota	Mice	2022	Li et al. (2022)
	Rats	2014	Cristofori et al. (2021)
		2014	Adak et al. (2014a)
		2017	Adak et al. (2014b)
		2022	Bai et al. (2022)
	Healthy males	2018	Karl et al. (2018)
	Mountaineers	2013	Kleessen et al. (2005) and Adak et al. (2013)
Study on plateau-related myocardial injury and intestinal microbiota	Rats	2022	Hu et al. (2022)
Study on plateau-related decrease in bone density and intestinal microbiota	Multi-ethnic Chinese cohort	2022	Zuo et al. (2022)
Study on plateau-related cognitive impairment and intestinal microbiota	Healthy college students	2011	Yan et al. (2011)
		2019	Ma et al. (2019)
	Mice	2022	Zhao et al. (2022)

#### 2.1.2 Changes in human intestinal microbiota after long-term exposure to high-altitude environments

**Figure 2:**
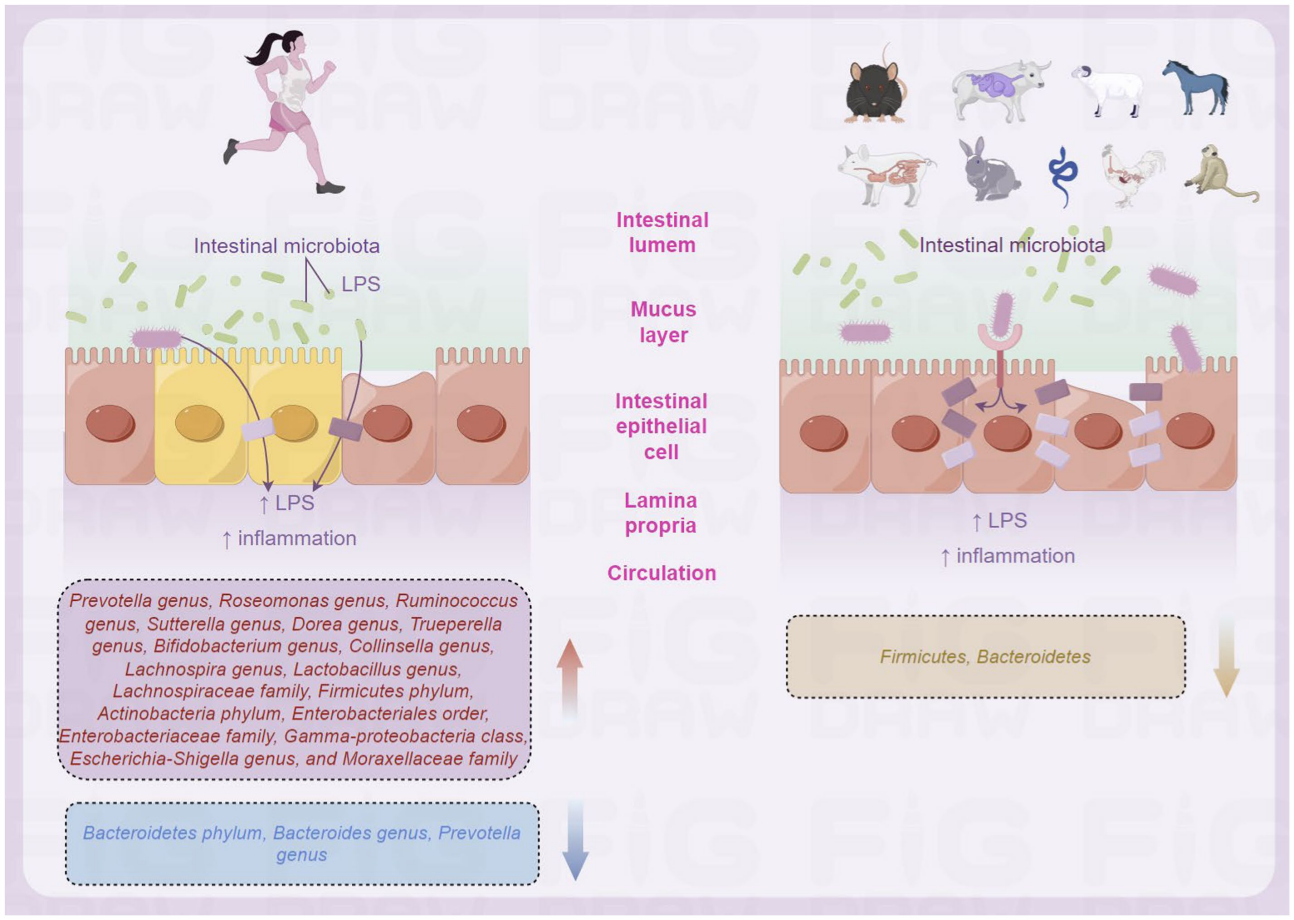
Differences in intestinal microbiota of populations in high-altitude (by Figdraw).

Previous studies have shown that the composition of intestinal microbiota in populations living in different regions varies due to unique living environments, genetic backgrounds, or dietary habits (Zhang et al., 2015, 2018; Yadav et al., 2016; Geng et al., 2023; Hu et al., 2023). An Indian study investigated the composition of intestinal microbiota in healthy adults living in high-altitude and low-altitude regions (das et al., 2018). Das et al. selected populations residing in Leh, the capital of Ladakh Province in northern India, and populations residing in Barabahal, Haryana Province, in northwestern India. The analysis showed that the intestinal microbiota of all healthy Indians was mainly composed of four bacterial phyla: Firmicutes (62%), Bacteroidetes (24%), Actinobacteria (5.2%), and Proteobacteria (4.2%) (das et al., 2018). The dominant bacterial genera were Prevotella, Bacteroides, Faecalibacterium, Roseburia, Ruminococcus, Lachnospira, Dialister, Trueperella, Bifidobacterium, Collinsella, Parabacteroides, and Enterobacteriaceae. In addition, although the observed bacterial abundance was low, the presence of members of the Verrucomicrobia, Lentisphaerae, and Synergistetes phyla was also detected in most subjects (das et al., 2018). When comparing the composition of intestinal microbiota between high-altitude and plain populations in India, it was found that the number of Bacteroidetes in the intestinal microbiota of high-altitude populations was higher, while the number of Proteobacteria was lower, and the intestinal microbiota of plain populations had a higher number of Firmicutes and Proteobacteria (das et al., 2018). Greenhill et al. found that significant differences in the number of selected bacterial phyla, including Bacteroidetes, Firmicutes, Enterobacteriaceae, Actinobacteria, Streptococcus, and total bacteria between high-altitude and plain populations, were found on the intestinal microbiota of populations living in high-altitude and plain regions of Papua New Guinea (Figure 2; Greenhill et al., 2015).

A study in China also found that high altitude may affect the composition of intestinal microbiota (Li and Zhao, 2015). The study selected three groups of subjects: Tibetans who permanently reside in Tibet (T group), Han Chinese who have lived in Tibet for more than 4 years (HH group), and populations living in plain areas (LH group) (Li and Zhao, 2015). After comparison, significant differences were found in the fecal microbiota composition among the three groups (Li and Zhao, 2015). The T and HH groups had a higher abundance of Firmicutes, while the LH group had a higher relative abundance of Bacteroidetes (Li and Zhao, 2015). The variation at the phylum level was related to the differences at the genus level, which was mainly attributed to a significant decrease in Prevotella in the HH group, a significant decrease in Bacteroides and Prevotella in the T group, and a significant increase in Lactobacillus in the T group (Li and Zhao, 2015). Another study in China found significant differences in the species composition of intestinal microbiota between Tibetans and Han Chinese living at the same altitude, between Han Chinese living at different altitudes, and between Tibetans living at different altitudes (Li et al., 2016). In Tibetans and Han Chinese, the relative abundance of Bacteroidetes and Firmicutes was the highest, accounting for more than 90% of all species. The relative abundance of Lentisphaerae, Actinobacteria, and Prevotella was higher in Tibetans than in Han Chinese, while the relative abundance of Proteobacteria, Fusobacteria, and Bacteroides was higher in Han Chinese than in Tibetans (Li et al., 2016). These results indicate that there are significant differences in the species composition of intestinal microbiota between Tibetans and Han Chinese, indicating the importance of genetic and cultural factors in the composition of intestinal microbiota (Li et al., 2016). Comparing the differences in intestinal microbiota between low-altitude Han Chinese and high-altitude immigrant Han Chinese populations, it was found that the number of Enterobacteriales, Enterobacteriaceae, γ-Proteobacteria, Escherichia/Shigella, and *Chromobacterium violaceum* in the intestinal microbiota of high-altitude immigrant Han Chinese was higher than that of low-altitude Han Chinese, indicating that different living environments also have a significant impact on intestinal microbiota (Li et al., 2016).

During the investigation of bacterial community structures in the intestinal microbiota of populations from different regions, researchers have identified that the composition of intestinal bacterial communities is driven by specific bacterial groups or genera. Consequently, the concept of “enterotypes” has been introduced, representing distinct microbial clusters (Arumugam et al., 2011). Different enterotypes can be distinguished by changes at the level of one of the following three bacterial genera: Bacteroides (enterotype 1), Prevotella (enterotype 2), and Ruminococcus (enterotype 3). Studies have found a negative correlation between the relative abundance of Bacteroides and Prevotella genera (Arumugam et al., 2011), where the relative abundance of Bacteroides is positively associated with diets rich in animal fat and protein, while the relative abundance of Prevotella is associated with diets low in animal fat and protein but high in carbohydrates and monosaccharides (Wu et al., 2011; Moeller et al., 2014). Therefore, some research suggests that individual differences in the composition of the human intestinal microbiota can be described using the ratio of Bacteroides to Prevotella genera (Costea et al., 2018).

After analyzing the intestinal microbiota of Tibetans and Han Chinese living long term in Lhasa (3,600 m), it was found that most Han Chinese belong to the Bacteroides enterotype, while most Tibetans belong to the Prevotella enterotype (Li et al., 2016). This may be attributed to the dietary structures of modern urban Han Chinese populations, which are characterized by low fiber, high animal protein, and high-fat diets, whereas Tibetan diets in high-altitude regions are more associated with high-fiber and high-carbohydrate consumption.

### 2.2 The changes in intestinal microbiota after acute high-altitude exposure

Acute mountain sickness (AMS) poses a significant risk, especially at altitudes above 2,500 m, where rapid ascent can lead to potentially fatal consequences, notably high-altitude cerebral edema (HACE) and high-altitude pulmonary edema (HAPE) (Liu et al., 2021). Alongside classic symptoms like headache, chest tightness, palpitations, and shortness of breath, AMS can also present gastrointestinal symptoms such as nausea, vomiting, and diarrhea. The disruption of intestinal microbiota may significantly contribute to the occurrence and severity of these gastrointestinal manifestations (Ma et al., 2019). A study of seven climbers who were examined for 47 days at an altitude of over 5,000 m found that Bifidobacterium decreased significantly, and bacterial species belonging to the genera of Pseudomonas, Corynebacterium, and Eggerthella also decreased significantly, while special Enterobacteriaceae bacteria with potential pathogenicity (such as *Escherichia coli*) increased (Kleessen et al., 2005). Furthermore, the increase in their quantity coincided with a decrease in serum IgM and/or IgA anti-LPS levels, while C-reactive protein (CRP) levels significantly increased post-high-altitude exposure (Li et al., 2022). These findings suggest that high-altitude hypoxic environments may alter the composition of intestinal microbiota and impact immunological parameters (Liu et al., 2021). An Indian study using *in vitro* bacterial culture methods found that the number of aerobic bacteria decreased significantly with the increase in anaerobic and facultative anaerobic bacteria during the adaptation of 15 soldiers to an altitude of 3,505 m for 15 days, thus proving that the low-oxygen environment of high altitude may change the composition of intestinal microbiota (Adak et al., 2013). In a randomized controlled trial involving the same cohort, participants resided for 21 days in low-altitude regions followed by a 22-day stay at a peak altitude of 4,300 m. Based on the occurrence of AMS, individuals were stratified into two groups (Karl et al., 2018). Analysis of fecal microbiota composition at low altitudes revealed that the AMS group exhibited a higher relative abundance of the Prevotella genus than the non-AMS group, indicating a lower ratio of Bacteroides to Prevotella genera in the AMS group (Karl et al., 2018). Moreover, individuals who did not experience a fat-free body weight loss exceeding 50% post-acute high-altitude exposure demonstrated a lower ratio of Bacteroides to Prevotella genera (Karl et al., 2018). This suggests the potential utility of the Bacteroides-to-Prevotella ratio as a biomarker for identifying individuals more susceptible to high-altitude effects (Zhang et al., 2018). Furthermore, irrespective of AMS symptoms, high-altitude weight loss correlated with a reduced relative abundance of Lactobacillus and Sutterella genera.

Animal experiments have found that mice exposed to an altitude of 5,000 m for 30 days and that exposure to high-altitude and low-oxygen environments did not change the relative abundance of aerobic, anaerobic, facultative anaerobic, potentially pathogenic Gram-negative bacteria, and Gram-positive bacteria in mice, but significantly reduced the relative abundance of Epsilonproteobacteria, Actinobacteria, Clostridia, and Spirochaetes, and increased the relative abundance of Verrucomicrobia, indicating that exposure to low oxygen and high altitude may affect the composition of intestinal microbiota (Zhang et al., 2018). In studies involving Wistar rats, it was observed that acute exposure to an altitude of 4,100 m resulted in a notable increase in the abundance of the Bacteroides genus, accompanied by a decrease in the abundance of the Prevotella genus, compared to the control group at sea level (Bai et al., 2022). This phenomenon indicates a potential adaptation of the intestinal microbiota to high-altitude environments through alterations in microbial composition. In a murine model of acute mountain sickness, hypoxia was found to modulate the composition of intestinal microbiota by promoting the secretion of the antimicrobial peptide angiogenin-4. Following hypoxia exposure, there was a significant decrease in the abundance of the Sutterella genus in the mouse intestine, while the abundance of the Desulfovibrio genus increased significantly (Zhao et al., 2022). Phospholipid metabolites from the Desulfovibrio genus are presented by intestinal epithelial CD1d, inducing the proliferation of IL-17A-producing γδT cells, thereby exacerbating intestinal damage (Li et al., 2022). Studies on rats acutely exposed to high-altitude environments have shown that hypoxic conditions at high altitudes lead to a decrease in the proportion of aerobic bacteria and an increase in the proportion of anaerobic bacteria. Among them, the phyla Firmicutes, Akkermansia genus, and Lactobacillus genus constitute the core microbial community under low-oxygen conditions. This suggests that high-altitude hypoxia is a significant environmental factor influencing the structure and diversity of intestinal microbiota, thereby impacting the homeostasis of the host intestinal environment (Zhang et al., 2018). Additionally, rats exposed to low pressure (429 mmHg) for 7 days exhibited a significant increase in the abundance of aerobic and facultative anaerobic bacteria in fecal samples, while the abundance of anaerobic bacteria significantly decreased. This indicates that atmospheric pressure is an important exogenous factor regulating the composition of intestinal microbiota (Bai et al., 2022).

## 3 Impact of intestinal microbiota alterations on organismal health

### 3.1 Effects of long-term high-altitude exposure on intestinal microbiota and organisms

The harsh conditions of high-altitude regions, characterized by low oxygen levels and extreme cold, necessitate increased energy intake to maintain body temperature, coupled with limited food availability. Studies have revealed that mammals and humans subjected to long-term high-altitude exposure exhibit a higher ratio and relative abundance of Firmicutes and Bacteroidetes (Geng et al., 2023). This unique microbial community specific to high-altitude species may possess enhanced capabilities to utilize high-fiber forage, aiding them in meeting their energy demands in cold and high-altitude habitats. This could contribute to maintaining intestinal homeostasis, energy equilibrium, and core body temperature in adverse environments (Duan et al., 2022). Furthermore, research confirms that the low-pressure and hypoxic conditions prevalent in high-altitude regions can induce inflammatory responses, leading to vascular leakage, accumulation of inflammatory cells in multiple organs, and elevated serum cytokine levels. Dietary intake of plant fibers has been shown to increase microbial diversity and reduce inflammatory markers (Zhao et al., 2023). Moreover, the collective metabolic activities of intestinal microbiota produce metabolites such as short-chain fatty acids (SCFAs), volatile fatty acids (VFAs), essential amino acids, and vitamins, aiding in the evolutionary adaptation of hosts to high-altitude environments (Geng et al., 2023). High-altitude mammals harbor abundant strains capable of producing SCFAs. For instance, plateau pikas and plateau pikas are rich in members of the families Lachnospiraceae and Clostridiaceae, which not only may enhance energy acquisition from food but also convert dietary fibers into SCFA (Wang et al., 2022). SCFAs primarily suppress inflammation by inhibiting the NF-κB pathway and/or histone deacetylase (HDAC) functionality, thereby downregulating pro-inflammatory cytokines (Hu et al., 2022). Similar phenomena have been observed in other high-altitude species. For example, a metabolomics study examining fecal samples from humans and pigs living at high and low altitudes revealed that the intestinal microbiota of individuals from high-altitude regions, including humans and Tibetan pigs, may produce more short-chain or long-chain fatty acids (Zeng et al., 2020).

**Figure 3:**
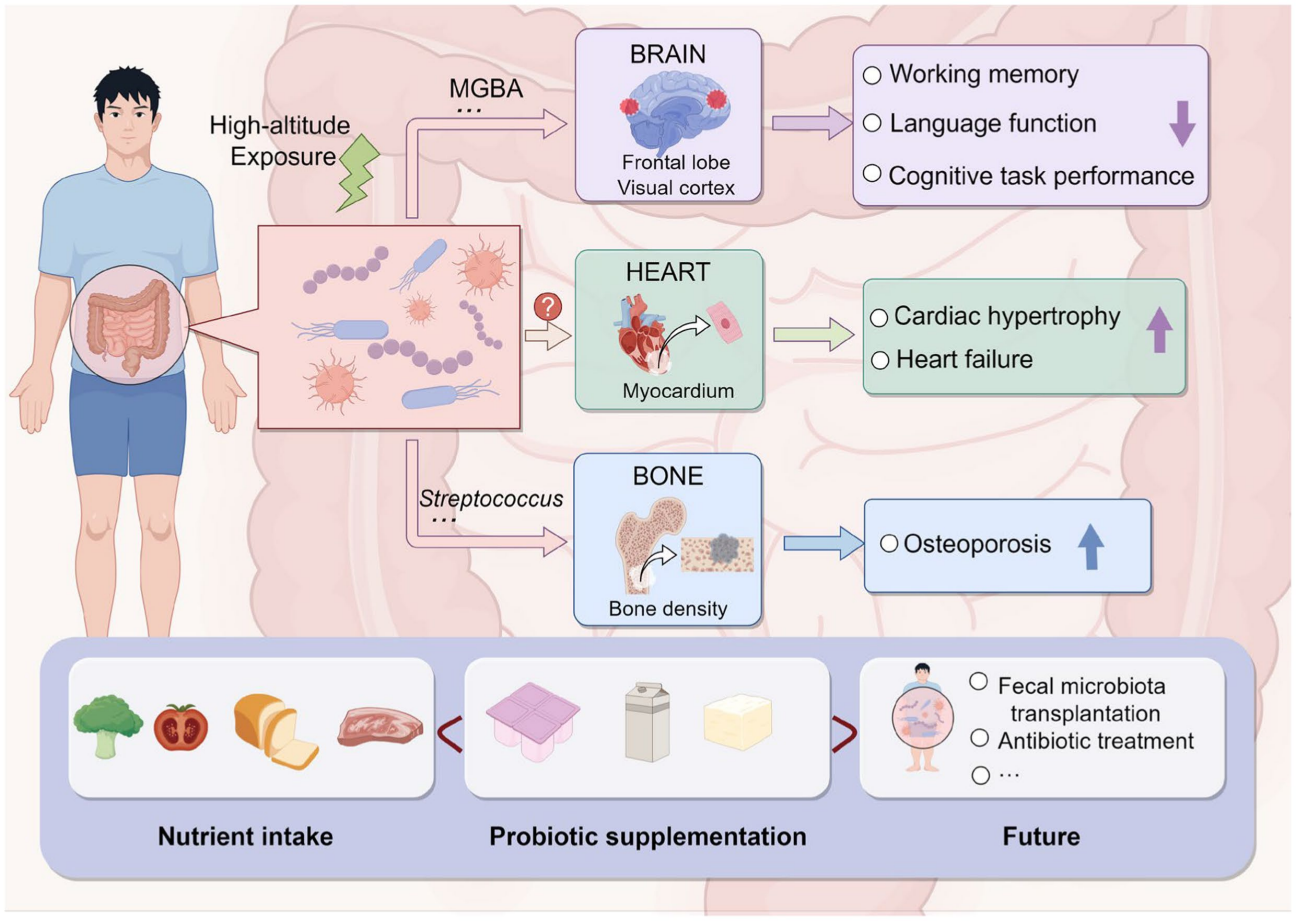
Impact of high-altitude-induced intestinal microbiota alteration on the human body and prevention strategies (by Figdraw).

In addition to affecting gastrointestinal function, high-altitude environments may also impact cardiac and other physiological functions (Figure 3). Loss of acclimatization to hypoxia in populations living in high-altitude regions can induce cardiac hypertrophy, leading to chronic heart failure and affecting cardiac output (Nakamura and Sadoshima, 2018). In addition to oxidative stress, inflammation, and kinase activity, altitude-related pathological cardiac hypertrophy following prolonged exposure to low-pressure hypoxia may also involve the intestinal microbiota, playing a crucial role in the pathogenesis and progression of cardiac enlargement and heart failure (Clyde, 2019). Moreover, a study on a rat model of high-altitude-related cardiac injury found that targeted modulation of intestinal microbiota can alleviate pathological cardiac hypertrophy and improve intestinal microbiota dysbiosis and metabolic changes induced by hypobaric hypoxia. This suggests that the intestinal microbiota may be a pathogenic factor and therapeutic intervention target for high-altitude-related myocardial injury (Hu et al., 2022). High-altitude environments may also affect bone density. A multicenter study conducted in China found a negative correlation between altitude and quantitative ultrasound index (QUI) of bones, with Streptococcus playing a significant mediating role, suggesting that high-altitude exposure may reduce adult bone density, thereby increasing the risk of osteoporosis, with potential involvement of the intestinal microbiota (Zuo et al., 2022).

### 3.2 Acute effects of high-altitude exposure on intestinal microbiota and organisms

The human intestinal microbiota, a complex and dynamic ecosystem, plays a crucial role in the maintenance of host health (Figure 3). A range of benefits are provided to the host by the intestinal microbiota, including regulation of the intestinal mucosal barrier, modulation of the immune system, and protection against invading pathogens. The composition and diversity of the intestinal microbiota are shaped by a range of factors, including diet, environment, and host genetics (Cani, 2012; Holmes et al., 2012; Hooper et al., 2012). The intestinal mucosal barrier represents a fundamental barrier that is primarily composed of tight junctions, gap junctions, mucins, immunoglobulins, intestinal flora, and other specialized immune cells. This barrier plays a crucial role in protecting the host from invading pathogens (Khanna et al., 2018). When acutely exposed to high altitudes is known to result in several physiological adaptations, including alterations in gastrointestinal function (Karl et al., 2013; Mackos et al., 2017), with consequences that may include the breakdown of intestinal mucosal barrier integrity leading to gastrointestinal dysfunction, an increase in channels for bacterial antigens such as lipopolysaccharide (LPS) to enter the circulation (Caesar et al., 2015), and increased susceptibility to disease and infection (van Wijck et al., 2012; Sharma and Riva, 2020). LPS is an endotoxin that can cause intestinal inflammation and increased intestinal tight junction permeability, leading to bacterial translocation and malnutrition, causing intestinal microbiota imbalance and many diseases (Cristofori et al., 2021; Bhatia et al., 2022).

The intestinal mucosal barrier represents a fundamental barrier that is primarily composed of tight junctions, gap junctions, mucins, immunoglobulins, intestinal flora, and other specialized immune cells. This barrier plays a crucial role in protecting the host from invading pathogens. However, a study on male albino rats has demonstrated that daily exposure to a simulated hypobaric hypoxia environment (4872.9 m, 55 KPa air pressure) for 8 h over a period of 30 days results in a significant reduction in aerobic bacterial density and a significant increase in anaerobic bacterial and *Escherichia coli* densities. These alterations in intestinal flora can lead to dysfunction of the intestinal mucosal barrier, which may not only cause small intestinal dysfunction but also disrupt the function of the large intestine mucosal barrier, ultimately resulting in systemic infection (Adak et al., 2014a,b). In addition, a study on male Wistar rats revealed that exposure to altitudes of 3,842 and 4,767 m for 3 days resulted in a significant increase in intestinal mucosal injury compared to the control group. Furthermore, the higher the altitude, the more severe the injury (Zhang et al., 2015). Notably, the expression of hypoxia-inducible factor 1α (HIF-1α) was significantly upregulated in the intestinal mucosa, which may be associated with intestinal mucosal barrier dysfunction (Zhang et al., 2015). The expression of HIF-1α and inducible nitric oxide synthase (iNOS) significantly increases with increasing hypobaric hypoxia exposure and may play a key role in intestinal mucosal injury (Zhang et al., 2015). Additionally, a study on Sprague Dawley rats found that exposure to a simulated hypobaric hypoxia environment at an altitude of 4,000 m for 3 days resulted in thinning of the intestinal mucosa and a reduction in epithelial cell numbers with irregular morphology under light microscopy (Luo et al., 2017). Furthermore, electron microscopy revealed damage to the intestinal epithelial cells, widening of the gaps between intestinal villi, the opening of tight junctions between cells, and decreased expression of the tight junction protein occludin. Collectively, these findings confirm that hypobaric hypoxia conditions can induce intestinal mucosal barrier injury (Luo et al., 2017). Studies on mountaineers have found that transient increases in gastrointestinal permeability are associated with an increased abundance of pro-inflammatory intestinal bacteria and inflammation (Kleessen et al., 2005). These findings suggest that the intestinal microbiota may be influenced by acute high-altitude exposure in hosts and play a role in the occurrence and progression of AMS.

Research on healthy university students in high-altitude regions has found that acute exposure to high altitudes can lead to decreased cognitive tasks and working memory; long-term exposure to high altitudes can also impair language and spatial working memory abilities in healthy university students (Ma et al., 2019), primarily manifested as reduced accuracy and response behavior, as well as decreased activity in certain brain regions, including the medial frontal lobe and visual cortex (Figure 3; Yan et al., 2011). Maintaining the balance of intestinal microbiota is considered an effective way to regulate cognitive abilities through the microbiome–intestinal–brain axis (MGBA) (Khanna et al., 2018). If the intestinal microbiota is disturbed, the intestinal environment may be affected, potentially leading to severe symptoms in the host. For example, damage to intestinal barrier integrity may allow bacteria and/or their metabolites from the lumen to enter the bloodstream, ultimately impairing brain function and cognitive abilities (Sharon et al., 2016). Studies on mice have also found that acute exposure to high altitudes for 14 days leads to working memory impairment, and subsequent exposure to high altitudes after antibiotic treatment exacerbates working memory impairment, suggesting that the MGBA may play an important role, with the family Helicobacteraceae being the most likely colonic bacterial family to affect cognitive impairment (Zhao et al., 2022).

In order to identify potential biomarkers within the intestinal microbiome that can predict responses to interventions or disease risks, some studies have proposed using the genera Bacteroides and Prevotella as potential biological markers related to diet and lifestyle. These markers may help predict individual responses to dietary interventions. Typically, a lower ratio of Bacteroides to Prevotella is considered indicative of a healthy, high-fiber, plant-rich diet. Additionally, Prevotella is known to produce SCFAs, and certain Prevotella bacteria have been associated with improved glucose homeostasis. However, it is important to note that Prevotella can also have detrimental effects due to environmental changes. Recent research suggests that during oxidative stress, Prevotella may rapidly proliferate, leading to disruption of intestinal mucosal barrier function and inflammation. Furthermore, some Prevotella species may even act as opportunistic pathogens. Meanwhile, intestinal microbiota dominated by Prevotella are specialized in degrading plant fibers, but this specialization reduces their fermentation potential for lipid breakdown and protein hydrolysis.

## 4 Strategies for managing potential alterations in intestinal microbiota following high-altitude exposure

### 4.1 Adjusting nutrient intake in the diet

The composition and activity of the intestinal microbiota are influenced by nutrient intake in the diet. Carbohydrate fermentation by intestinal microbiota leads to the production of SCFAs such as acetic acid, propionic acid, and butyric acid (Macfarlane and Macfarlane, 2012; Verbeke et al., 2015). SCFAs (especially butyric acid) are beneficial for gastrointestinal health, but other amino acid fermentation products (such as ammonia) may damage the intestinal mucosal barrier (Verbeke et al., 2015). Consuming a high-protein, a high-fat diet (>40% of total intake), and a low-carbohydrate (<20% of total intake), low-fiber diet reduces the concentration of beneficial intestinal bacteria SCFAs in feces and increases the concentration of amino acid metabolites (David et al., 2014; O’Keefe et al., 2015). Studies in non-high-altitude areas have shown that although a high-protein diet can increase the concentration of amino acid metabolites in feces, it does not affect the composition of intestinal microbiota and gastrointestinal health, indicating that protein fermentation does not seem to seriously reduce the function of the intestinal mucosal barrier (Smith et al., 2013; Beaumont et al., 2017). However, the low-pressure and low-oxygen environment of high altitudes may make the intestinal mucosal barrier more sensitive to intestinal bacteria and their metabolites (Tsuboi et al., 2020). At the same time, the low ratio of Bacteroides/Prevotella may be related to the occurrence of AMS, and its possible mechanism is insufficient fermentation potential for fat decomposition and protein hydrolysis (Šuligoj et al., 2020). Therefore, a high-carbohydrate, high-fiber, low-protein, and low-fat diet is recommended for acute high-altitude exposure to reduce the incidence of AMS.

### 4.2 Supplementation of probiotics

Although it is not yet clear to what extent the intestinal microbiota is beneficial or harmful, it is currently believed that the Staphylococcus and Veillonella genera have potential hazards, while the Bifidobacterium, Streptococcus, Bacteroides, Prevotella, Collinsella, and Clostridium genera of intestinal symbiotic bacteria contain both potential benefits and harms (Tachie et al., 2024). However, some classifications in the intestinal microbiota are generally considered beneficial, called probiotics, which are defined as active microorganisms that, when applied in appropriate doses, have beneficial effects on the host’s health. The most classic examples are the Lactobacillus and Bifidobacterium genera. Strains in these genera can enhance the host’s immune function, secrete compounds that aid digestion, prevent the colonization of pathogens, and regulate gastrointestinal function (Hill et al., 2014). Recent studies suggest that strains in the Actinomyces, Roseburia, and Faecalibacterium genera may also be probiotics (Gibson et al., 2017). These strains can produce butyric acid in SCFAs, enhance the integrity of the intestinal mucosal barrier, reduce inflammation and oxidative stress, and thus affect the health of the intestinal and beyond (Canani et al., 2011). In a study of the daily diet of Tibetans, it was found that yak milk and its dairy products, which are the main part of the Tibetan diet, have a higher average number of lactic acid bacteria than yogurt made from cow’s milk, and the number of yeast strains is also higher than that of cow’s yogurt. The content of coliforms (fecal contamination or pathogenic microorganisms in the intestine) is significantly lower than that of cow’s yogurt, which may be related to the adaptation of Tibetans to the high-altitude environment and the relief of AMS (Guo et al., 2014). Therefore, supplementing probiotics with food and medicine may be beneficial for preventing and alleviating AMS after high-altitude exposure.

## 5 Conclusion

The high-altitude environment poses significant challenges to human adaptation. However, with the rise of tourism and education industries, there has been a dramatic increase in the number of people visiting high-altitude regions annually. At high altitudes, the oxygen content is only 50–60% of that at sea level. As individuals ascend from low-altitude to high-altitude areas, the partial pressure of oxygen gradually decreases, leading to hypoxic reactions and severe damage to various organs, with gastrointestinal damage being particularly prominent (Geng et al., 2023). Gastrointestinal stress response is a common manifestation of acute altitude sickness, characterized by pronounced gastrointestinal discomfort such as diarrhea, nausea, vomiting, and anorexia (Duan et al., 2022). The occurrence of acute gastrointestinal diseases at high altitudes is directly related to the disruption of digestive function induced by hypoxia. However, the specific mechanisms underlying the changes and damage to the digestive system under hypoxic conditions are not yet fully understood, particularly regarding the impact on gastrointestinal mucosal damage, indicating a need for further investigation and attention.

The intestine harbors a diverse microbiota, comprising over 400 species of bacteria, with approximately 99% being obligate anaerobes (Hu et al., 2023). The intestinal microbiota forms a mutualistic ecosystem, crucial for human health, constituting the intestinal barrier. The intestinal microbiota includes aerobic, anaerobic, and facultative bacteria (Wang et al., 2022). It has been established that obligate anaerobes, primarily Bifidobacteria, colonize the mucosal epithelial surface, forming a biofilm that impedes the adhesion and colonization of potentially pathogenic bacteria such as *Escherichia coli*, exhibiting “colonization resistance.” Disruption of the intestinal microbiota, due to various factors, may lead to the proliferation of opportunistic bacteria (mainly *Escherichia coli*, *Klebsiella* spp., and *Enterococcus*), which can breach the damaged biofilm and mucosal barrier, a phenomenon known as bacterial translocation (Liu et al., 2021; Hu et al., 2022). Bacterial translocation occurs horizontally, from the original site to adjacent regions, and vertically from the mucosa to deeper layers and systemic circulation, resulting in systemic infection. Rapid exposure to high-altitude hypoxic environments induces prolonged stress responses in the body, leading to increased sympathetic nervous system activity, vasodilation in the gastric and duodenal mucosa, decreased blood flow, exacerbating mucosal ischemia and hypoxia, ultimately causing mucosal damage and increased permeability (Yan et al., 2011; Ma et al., 2019). Experimental evidence from high-altitude studies indicates significant differences in inflammatory mediators between individuals acclimated to high altitudes and those from low-altitude regions, where nitric oxide, tumor necrosis factor, and reactive oxygen species contribute to mucosal injury and increased permeability (Ma et al., 2019; Du et al., 2022; Wang et al., 2022; Zhao et al., 2022, 2023). The mechanisms may involve apoptosis induction in epithelial cells, disruption of intercellular tight junctions by tumor necrosis factor, and lipid peroxidation in intestinal epithelial cell membranes mediated by reactive oxygen species (Ma et al., 2019; Du et al., 2022; Wang et al., 2022; Zhao et al., 2022, 2023). The compromised intestinal barrier function destabilizes the internal environment, facilitating bacterial translocation. When intestinal permeability increases sufficiently, macromolecules, bacteria, and lipopolysaccharides can breach the damaged mucosal barrier, leading to tissue invasion and bacterial translocation, culminating in intestinal endotoxemia, which can be solved by fecal microbiota transplantation and antibiotic treatment (Karl et al., 2018; Cristofori et al., 2021). Intestinal endotoxemia exacerbates mucosal damage, further promoting bacterial translocation and establishing a vicious cycle. The described mechanisms contribute to bacterial translocation and subsequent consequences, forming a vicious cycle. However, the mechanisms underlying the alteration of the intestinal barrier in response to high-altitude hypoxia remain incompletely elucidated, necessitating further investigation.

The human intestinal microbiota is closely associated with immunity, hormone regulation, metabolic homeostasis, various diseases, and specific environments (Guo et al., 2021; Liu et al., 2021). It serves as a vital indicator of human adaptation to different environments. The composition of the intestinal microbiota varies among individuals residing at different altitudes (Tandon et al., 2018; Clyde, 2019; Liu et al., 2021). Environmental influences on the intestinal microbiota have been demonstrated in both animals and humans.

Nevertheless, the specific changes in intestinal microbiota among populations at different altitudes and among different ethnic groups at high altitudes, the pathways and mechanisms through which the high-altitude environment affects the human intestinal microbiota, and its potential association with altitude sickness, require further investigation. Additionally, exploring more effective strategies to prevent imbalances in the intestinal microbiota at high altitudes is necessary. Research on the intestinal microbiota in high-altitude environments provides insights into the mechanisms underlying the effects of high altitudes on human health, offering new perspectives and approaches for the prevention and treatment of altitude-related diseases. Future studies should expand to collect fecal samples from larger populations at various altitudes, utilizing high-throughput sequencing and other biotechnological tools to analyze the structure and diversity of the intestinal microbiota, elucidating the effects of high-altitude environments and their mechanisms. Subsequently, investigating the role of the intestinal microbiota in the occurrence and development of high-altitude diseases, exploring whether intestinal microbiota dysbiosis can serve as an early warning indicator of altitude sickness, and assessing the potential of modulating intestinal microbiota structure and function for the prevention and treatment of altitude-related diseases are essential. Finally, intervening in the intestinal microbiota through diet, probiotics, and other means, observing changes in intestinal microbiota structure and function and their effects on human health, provides a scientific basis for developing nutrition and health products suitable for high-altitude environments, which is significant for both indigenous high-altitude residents and newcomers to high altitudes.

## Author contributions

DL: Investigation, Writing – original draft. DC: Conceptualization, Funding acquisition, Project administration, Writing – review & editing. JX: Writing – review & editing, Data curation, Investigation. WW: Investigation, Methodology, Writing – original draft. L-JZ: Investigation, Validation, Writing – review & editing. HP: Investigation, Writing – review & editing, Resources. CH: Writing – review & editing, Conceptualization, Funding acquisition, Supervision. HY: Conceptualization, Funding acquisition, Supervision, Writing – review & editing.
